# A step further towards scientific arguments for expert witnesses in the defense of patients accused of forensic parasomnias

**DOI:** 10.1093/sleep/zsae042

**Published:** 2024-06-21

**Authors:** Geert Mayer

**Affiliations:** Adjunct professor of Philipps-Universität Marburg, Department of Neurology, Marburg, Germany

Whoever sat in court to defend a patient accused for sexual assault or violence during sleep knows about the paucity of objective and scientific arguments for a sleep expert witness that often stands against the conviction of legal and public morals. The article by Peretti et al. [1] provides new important arguments that help the expert witness to clarify if the assaults under accusation are caused by a disorder of arousal (DOA) with or without sexsomnia or if the latter is pretended. Apart from these arguments, public opinion needs to get a different view on this deviant behavior, which is not intentional, but occurs in a state of blurred/lack of awareness/consciousness due to a dissociated state between wake and sleep. The judges and the jury must understand and be convinced that an underlying sleep disorder may be the cause. The task of sleep experts is to prove the sleep disorder with strong scientific arguments.

Recently Pressman [2] addressed the dilemma of retrospectively proving a DOA. He focused on amnesia during the respective criminal act by going through selected cases of forensic cases. Since there are no standard procedures to assess cognition “A recommended method is the construction of a detailed chronology of the episode bringing in evidence from the defendant, the victim, and any other witnesses.” The author argues that the absence of one or several proven executive functions (like planning etc.) plus dissociative analgesia are strong arguments for the presence of a DOA, which however, “have to be weighed against other arguments.”

DOAs can occur during sleep stage N2, but mainly during sleep stage N3. The timing of a criminal act during suspected DOA is therefore of major importance. Although latencies to N3 are considered to occur in the first third of the night, there is ample evidence that DOA onset is earlier. Pressman [3] reviewed 26 articles on DOA in which full polysomnography (PSG) was performed. These were categorized into DOA from sleep stage N2, from sleep stage N3, effects of sleep deprivation, and effects of alcohol on latency from sleep onset to slow wave sleep (SWS) in normal sleepers. Lowest SWS latency in 24-hour sleep-deprived healthy persons was 12.4 ± 1.8 minutes. The effect of a combination of sleep deprivation plus alcohol on latency of SWS in normal sleepers was much larger than that of sleep deprivation only. The SWS latencies in three studies on DOA patients and in one forensic case ranged from 9.7 to 28 minutes.

What did Peretti et al. [1] do to improve diagnostic issues in this medical and legal issue? In a retrospective study, the authors investigated latencies of first and last N3 interruptions in 47 patients with DOA with sexsomnia and 117 without sexsomnia (nsDOA). Patients with forensic issues were excluded, whereas those with comorbid neurologic diseases, mild sleep disorders, and medications were included. Patients underwent a medical interview, had two nights of video-PSG, performed the Epworth Sleepiness Scale and the Paris Parasomnia Severity Scale (PADSS). Demographic data revealed major differences between both groups: sexsomnia patients had less often a history of DOA than nsDOA and had their first episodes during adulthood. They had less frequently sleepwalking, sleep terrors and confusional arousals, they had less frequently injured themselves and others and had lower scores on the Epworth Sleepiness Scale. In video-PSG, sexsomnia patients had less N3 interruptions associated with complex behavior on both nights (47.9%, 51.2%) compared to nsDOA patients (79.7%, 80%). In both nights and both groups latencies from sleep onset to N3 interruptions with complex behavior were longer (median 56 and 57 minutes) than latencies to first N3 interruption (median 31 and 36 minutes). On the second night, 52.4% had complex behavior a few minutes after N3 interruption with simple behavior and 18.6% had their first N3 interruption with complex behavior. EEG analysis revealed shorter latencies to N3 interruption with fast EEG activity from sleep onset than to N3 interruptions with slow/mixed EEG activity.

Until recently typical pathological changes in PSG performed after forensic parasomnias could not be discovered and therefore were not helpful [4]. Analyzing video-PSG Lopez et al. [5] described EEG patterns that revealed different kinds of “dissociated awakenings,” depending on the change of EEG patterns from SWS into different kinds of sleepwalking behavior. According to the latter, those of Peretti’s cases of N3 interruption with fast EEG activity and complex behavior should be the ones with the most elaborate behavior, whereas the majority of cases with intermediate EEG activity should only show little elaborate motor activity.

In conclusion, Peretti et al. add important PSG-based arguments to the defense of forensic parasomnia, especially to the differentiation of DOA and DOA with sexsomnia. Despite these important findings, the limitations are that their PSGs have not been performed in forensic cases, in real-life settings, or in similar conditions as in the criminal act. Of note, the authors realize how difficult it is to assess the time of sleep onset retrospectively, which is essential for the latency to N3 interruption.

Peretti et al. findings have to be added to the array of arguments from Pressman, Lopez, and Espa [6], who described fragmentation of the first sleep cycles with consecutive delayed built up and decay of SWS in the course of the night. Taken into account the frequent parasomnia triggers are alcohol consumption and/or sleep deprivation, N3 latencies to complex behavior could be even shorter than the ones observed by Peretti et al.
